# Urethra-Sparing Robot-Assisted Simple Prostatectomy for Postoperative Antegrade Ejaculation

**DOI:** 10.3390/jcm12144867

**Published:** 2023-07-24

**Authors:** Sae Woong Choi, Dong Wan Sohn, U-Syn Ha, Sung-Hoo Hong, Ji Youl Lee, Hyuk Jin Cho

**Affiliations:** 1 Department of Urology, Yeouido St. Mary’s Hospital, College of Medicine, The Catholic University of Korea, Seoul 07345, Republic of Korea; lifeisa9ame@catholic.ac.kr (S.W.C.); druroking@catholic.ac.kr (D.W.S.); 2 Department of Urology, Seoul St. Mary’s Hospital, College of Medicine, The Catholic University of Korea, Seoul 06591, Republic of Korea; ushamd@catholic.ac.kr (U.-S.H.); toomey@catholic.ac.kr (S.-H.H.); uroljy@catholic.ac.kr (J.Y.L.)

**Keywords:** benign prostatic hyperplasia, robotic surgery, simple prostatectomy, ejaculation function

## Abstract

Background: We report a comparative analysis of extraperitoneal urethra-sparing robot-assisted simple prostatectomy (EUS-RASP) versus robot-assisted simple prostatectomy (RASP) using the Freyer approach for patients with a large prostate volume greater than 80 mL. Methods: A total of 32 patients underwent EUS-RASP, and 30 underwent RASP from April 2018 to November 2021. All the perioperative data and 6-month follow-up data were collected prospectively. We retrospectively evaluated baseline characteristics and functional outcomes, including International Prostate Symptom Scores (IPSSs) and quality of life (QOL), maximum flow rate, and post-void residual volume, between the two groups. Sexual function was analyzed in the EUS-RASP group. Results: The patients undergoing EUS-RASP and RASP had comparable baseline characteristics and functional outcomes. The EUS-RASP group showed a shorter operative time (123.4 ± 15.2 min vs. 133.7 ± 21.4 min, *p* = 0.034), length of hospital stay (2.9 ± 1.5 days vs. 4.6 ± 1.5 days, *p* = 0.001), and catheterization time (2.4 ± 1.7 days vs. 8.1 ± 2.4 days, *p* < 0.001). A total of 14/32 (43.8%) patients reported normal preoperative ejaculatory function in the EUS-RASP group, and 11/14 (78.6%) maintained antegrade ejaculation postoperatively. Conclusions: Extraperitoneal urethra-sparing RASP is an effective and feasible procedure that can improve voiding function and allow for the maintenance of ejaculatory function in patients with large prostates.

## 1. Introduction

Robot-assisted simple prostatectomy (RASP) was first introduced in 2008, during the widespread introduction of robotic techniques into urological surgery [1]. Different approaches using robotic systems have been developed since then and encouraging outcomes of RASP versus open simple prostatectomy (OSP) have been reported [2,3,4]. A variety of robotic surgical techniques, including transperitoneal and extraperitoneal approaches, transcapsular (Milin procedure), transverse vesicocapsular (Freyer procedure), and longitudinal vesical (modified Freyer procedure) incisions, and urethra-sparing (Medigan procedure) techniques based on the traditional OSP procedure, have been introduced [1,5,6,7,8,9,10,11,12]. Although RASP has become widely adopted as a surgical treatment for large benign prostatic hyperplasia (BPH), some problems remain unsolved, such as postoperative complications, continuous bladder irrigation (CBI), cost issues, and sexual dysfunction. Among these, a surgical treatment method that could reduce retrograde ejaculation should be considered for sexually active men.

We considered the urethra-sparing RASP (US-RASP) approach as a promising solution among several attempts to address these concerns. Due to technical challenges in urethra-sparing prostatectomy, US-RASP has been attempted relatively recently, but only a few studies have been reported [11,12,13,14]. We evaluated the feasibility of extraperitoneal urethra-sparing RASP (EUS-RASP) and compared the perioperative outcomes between EUS-RASP and Freyer approach RASP.

## 2. Materials and Methods

This study was a retrospective review of data prospectively collected into an Institutional Review Board (IRB)-approved registry database (IRB approval number SC22RISI0059). Consecutive patients with large BPH (>80 mL) underwent EUS-RASP and RASP (Freyer approach) from April 2018 to November 2021. All the perioperative and functional data and at least 6 months of follow-up data were collected prospectively. The primary purpose of this study was to introduce our EUS-RASP technique and evaluate its perioperative and functional outcomes.

### 2.1. Study Design

The inclusion criteria were (1) patients with BPH of more than 80 mL measured by transrectal ultrasound (TRUS) and the failure of medical treatment and (2) those with the ability to understand the details of a validated questionnaire, including the International Prostate Symptom Score (IPSS). The IPSS (and quality of life score, QOL) was administered to all the patients preoperatively and 6 months postoperatively. Sexual function was assessed in the EUS-RASP cohorts before surgery and 6 months postoperatively using the International Index of Erectile Function-5 (IIEF-5) and the Male Sexual Health Questionnaire-Ejaculation Dysfunction-Short Form (MSHQ-EjD-SF).

In all cases, multiparametric magnetic resonance imaging (mpMRI) was performed to evaluate anatomical structures in the pelvic cavity and for prostate cancer screening before surgery. In the cases with high prostate-specific antigen levels (PSA, ≥4 ng/mL), abnormal digital rectal examination, or definite focal mass lesion suggestive of malignancy in the prostate gland on mpMRI or TRUS, the patients underwent a TRUS-guided or target biopsy to exclude the presence of prostate cancer. Patients with a median prostate lobe were excluded from the EUS-RASP group. A single experienced urologist performed the EUS-RASP for 32 patients. Another experienced urologist performed a Freyer procedure RASP for 30 patients.

### 2.2. Surgical Techniques

All the EUS-RASPs and RASPs were performed using a da Vinci Xi Surgical System (Intuitive Surgical-ISRG, Sunnyvale, CA, USA) with a four-arm configuration.

#### 2.2.1. EUS-RASP Technique

(1) Preparation for robot docking via the extraperitoneal approach and technique to place trocars

**Figure 1:**
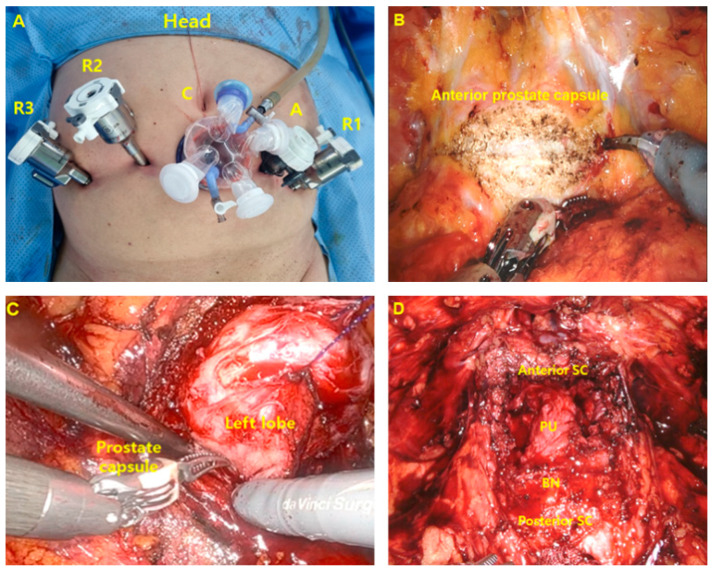
(**A**) Port placement for extraperitoneal urethra-sparing robot-assisted simple prostatectomy. R1, R2, and R3: 8 mm robot arms, C: glove port for robot camera, A: 10-mm assistant port. (**B**) A transverse incision was made on the anterior prostate capsule. (**C**) Adenoma enucleation was performed following the avascular plane of the surgical capsule, starting from the left lobe. (**D**) Status after removal of prostatic adenoma. SC: surgical capsule, PU: prostatic urethra, BN: bladder neck.

Under general anesthesia, the patient was placed in the Trendelendurg position (about 10–15 degrees) in a supine state. Sliding was prevented by the placement of shoulder support. An indwelling urethral catheter (16 Fr, 2-way) was inserted after sterilizing the surgical site and preparing the surgical instruments. After making a 3 cm vertical incision 1.5–2 cm below the umbilicus, subcutaneous tissues and Scarpa’s fascia were dissected. After the right rectus sheath was incised longitudinally, the rectus abdominis muscle and the peritoneum were bluntly separated with a finger while the rectus muscle was pushed laterally. When adequate space was created, the preperitoneal distention balloon system (PDB; Covidien Co., Ltd., MN, USA) was inserted toward the symphysis, and the balloon was fully inflated 2 or 3 times to secure the extraperitoneal surgical space as much as possible. After removing the PDB balloon system, a glove port (Nelis, Seoul, Republic of Korea) was inserted. Confirming that it was sealed, gas was injected to form a pneumopreperitoneum. The robot camera port was placed in the midline incision area with the peritoneum uninjured. The remaining robot trocars and an assistant port (three 8-mm robot instruments and one 10-mm trocar for assistance) were placed, as shown in Figure 1A. The robot trocars were positioned at least 6 cm apart, with the assistant port placed between the camera and the left robot trocar for convenience. Robotic instruments: 30° lens; monopolar curved scissors and needle driver on the right arm; bipolar forceps on the left arm; ProGrasp forceps on the fourth arm.

(2) Urethra-sparing adenoma enucleation method in the retropubic space

After exposing the prostatic capsule by removing pre-prostatic fat from the retropubic space, a transversal incision was made halfway between the bladder neck and the dorsal venous complex (Figure 1B). The cleavage plane between the surgical capsule and the adenoma is identified anteriorly. For proper traction of the adenoma, a 3-0 absorbable suture was added and then pulled adequately using robotic ProGrasp forceps (Figure 1C). Dissection was performed sequentially from the most exposed base side to the apex side. Medial side dissection was performed last, and delicate dissections were used to avoid urethral injury. The adenoma could be removed by dividing it into several pieces rather than trying to remove it in one piece to improve the view of the surgical field and dissect the surgical plane accurately. The same steps were repeated contralaterally. When it was judged that the adenoma of the surgical margin was sufficiently removed, the bladder was filled (100–150 cc) with normal saline to check for urethral injury (Figure 1D). If there was minor urethral tearing, it was repaired with 4-0 absorbable sutures. Thereafter, 3-0 or 4-0 absorbable sutures were used to provide additional bleeding control, and the prostate capsule was closed using 3-0 barbed sutures (Monofix, Samyang Holdings Corp., Seoul, Republic of Korea). Hemostatic products were applied to the incision site, and a surgical drainage was placed. After placing the excised prostate adenoma tissues into a surgical bag and removing it through the midline incision, the wound was closed layer by layer.

(3) Removing concomitant bladder stones

If bladder stones were present, they were removed after prostatic adenoma removal. After opening the anterior wall of the bladder longitudinally, the stones were removed from the bladder and placed in the surgical bag with the prostate adenoma. The bladder incision was closed with watertight 3-0 absorbable sutures. Finally, the bladder was filled with 150 cc of saline to check for leaks. In the case of a concomitant bladder stone, the indwelling urethral catheter was removed on the fifth day after surgery.

#### 2.2.2. RASP Using the Freyer Procedure

**Figure 2:**
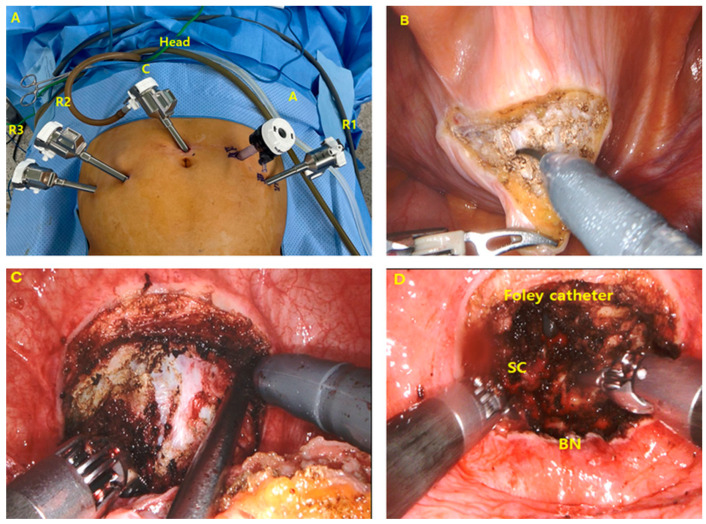
(**A**) Port placement for robot-assisted simple prostatectomy using the Freyer approach. R1, R2, and R3: 8 mm robot arms, C: port for robot camera, A: 10-mm assistant port. (**B**) Transverse incision at the dome of the bladder. (**C**) The bladder mucosa was incised, and dissection was carried distally to the apex. (**D**) Status after removal of prostatic adenoma. SC: surgical capsule, BN: bladder neck.

The technique has been described in detail in a previous paper [9] and is briefly reported here. The patient was positioned in lithotomy and steep Trendelenburg, and a transperitoneal approach with a five-port configuration was used, identical to that used in US-RASP (Figure 2A). After pneumoperitoneum, the bladder was filled with 300 mL of saline through an indwelling catheter. A transverse cystotomy was made just below the dome of the bladder, exposing the prostate base and trigone (Figure 2B). A circumferential incision was made in the mucosa covering the adenoma near the bladder neck. The incision was deepened to reveal an avascular plane between the adenoma and the prostatic capsule. The dissection then progressed circumferentially, sweeping laterally and anteriorly (Figure 2C). To facilitate exposure, a 3-0 absorbable suture was placed through the adenoma to provide traction with the ProGrasp forceps. The dissection was extended to the prostatic apex to expose the urethra at the apex, and the urethra was then divided under direct vision. After ensuring effective hemostasis with bipolar coagulation, the next step involved trigonization of the bladder neck (Figure 2D). This was accomplished by using a running suture technique with 3-0 barbed sutures, which seamlessly connected the bladder neck mucosa to the prostatic urethra. A 22 Fr three-way catheter was placed with the balloon filled in the prostatic fossa, and the cystotomy was closed with a running suture of 3-0 barbed sutures. A leak test was performed to confirm watertight closure. CBI was then initiated. Finally, a drain was placed in the rectovesical pouch, followed by extraction of the specimen and closure of the fascia and skin.

#### 2.2.3. Perioperative Data

The initial clinical workup data, including detailed history-taking and physical examination, age, body mass index (BMI), American Society of Anesthesiologists (ASA) score, laboratory tests, and prostate volume measured by TRUS, were gathered. All the patients underwent IPSS and QOL evaluations and measurements of PSA levels, maximal urinary flow rate (Qmax), and post-void residual volume (PVR) preoperatively and 6 months postoperatively. Other postoperative data included operative time, pathological adenoma weight, the presence of CBI, CBI duration, time to urethral catheter removal, time to drain removal, and length of stay. Postoperative complications were assessed according to the Clavien–Dindo classification system [15]. 

#### 2.2.4. Postoperative Management 

After surgery, a 22 Fr 3-way Foley catheter was placed, and CBI was performed in the RASP patients. CBI was not performed in the EUS-RASP group, and the Foley catheter was removed the day before or on the day of discharge. If significant hematuria persists, CBI is prolonged. The Foley catheter was removed on the seventh day after discharge in the RASP group or on the day of discharge only for patients hospitalized for about one week. Prophylactic antibiotics with second-generation cephalosporins were administered from the day of surgery to the first postoperative day. Both groups were encouraged to ambulate early.

#### 2.2.5. Statistical Analysis

The categorical variables are reported as frequencies and percentages using the chi-squared and Fisher’s exact tests. The continuous variables are reported as the means with standard deviation using the Student’s *t*-test. Statistical significance was indicated by a two-sided *p*-value of < 0.05. All the statistical tests were performed with the Statistical Package of Social Science for Windows (SPSS, Inc., Chicago, IL, USA), version 22.0.

## 3. Results

The patient characteristics and BPH-related complications are summarized in Table 1. There were no differences in the age, BMI, ASA scores, preoperative PSA levels, or prostate volume between the EUS-RASP (n = 32) and RASP (n = 30) groups. The BPH-related complications before surgery between the two cohorts (43.8% vs. 56.7%, *p* = 0.446) were not significantly different. 

Table 1 shows the perioperative outcomes and postoperative complications. The mean operative time was shorter in the EUS-RASP group (123.4 ± 15.2 min vs. 133.7 ± 21.4 min, *p* = 0.034). The amount of estimated blood loss, blood transfusion rate, and change in serum hemoglobin levels were not significantly different between the groups. Both groups showed a similar resected adenoma weight (59.4 ± 21.0 g vs. 57.7 ± 21.7 g, *p* = 0.765), postoperative PSA levels (1.1 ± 1.1 ng/mL vs. 1.0 ± 1.0 ng/mL, *p* = 0.882), and follow-up duration (9.9 ± 3.1 months vs. 9.3 ± 3.1 months, *p* = 0.496). The time to Foley catheter removal (1.9 ± 0.6 days vs. 8.1 ± 2.4 days, *p* < 0.001) and length of hospital stay (2.5 ± 0.7 days vs. 4.6 ± 1.5 days, *p* < 0.001) were significantly longer in the RASP group where CBI was not performed. No patient in the EUS-RASP group reported urinary incontinence, whereas one (3.3%) patient in the RASP group did. There was no postoperative urethral stricture in either group. 

In the EUS-RASP group, four cases with a wide-opened urethra injury on both sides were converted to conventional RASP and were excluded from the study. Six (6/32, 18.8%) cases of minimal urethral tearing were sutured with 4-0 monofilament stitches. Complete urethra-sparing simple prostatectomy without any urethral tearing was performed in 26/32 cases (81.3%). 

There was no significant difference in the rate of postoperative complications (12.5% in the EUS-RASP cohort vs. 16.7% in the RASP cohort, *p* = 0.728). In the EUS-RASP cohort, one (3.1%) patient experienced wound infection (grade 1), and three (10.0%) patients had grade 2 postoperative complications (urinary tract infection, mild hematuria after urethral catheter removal, and delirium, respectively). In the RASP cohort, three (10.0%) patients had grade 2 postoperative complications (two postoperative blood transfusions and mild hematuria after catheter removal, respectively), and two patients experienced grade 3a postoperative complications (two right inguinal hernias (RIH)). One patient who experienced RIH had anal pain after urination for several days postoperatively. 

The pre- and postoperative functional outcomes are presented in Table 2. The baseline IPSSs (22.3 ± 6.8 vs. 23.8 ± 6.1, *p* = 0.369), IPSS-QOL (6.4 ± 2.6 vs. 7.2 ± 2.8, *p* = 0.226), Qmax (6.6 ± 3.9 mL/s vs. 5.9 ± 3 mL/s, *p* = 0.439), or PVR (141.8 ± 155.8 mL vs. 153.5 ± 135.4 mL, *p* = 0.753) showed no statistically significant differences between the two groups. At the 6-month follow-up, there were significant improvements in the IPSSs (6.4 ± 2.6 vs. 7.2 ± 2.8, *p* = 0.252), IPSS-QOL (1.5 ± 1.1 vs. 1.7 ± 1.0, *p* = 0.433), Qmax (19.4 ± 6.4 mL/s vs. 17.1 ± 6.0 mL/s, *p* = 0.159), and PVR (11.1 ± 9.2 mL vs. 16.9 ± 16.9 mL, *p* = 0.104) in the two groups. 

In the EUS-RASP group, 18 patients (18/32, 56.3%) were sexually active (IIEF-5 > 17), and 14 of those patients (14/32, 43.8%) had normal ejaculatory function preoperatively. All the sexually active patients maintained sexual potency postoperatively (preoperative IIEF-5 vs. postoperative IIEF-5; 20.4 ± 2.1 vs. 20.1 ± 2.1). Eleven (11/14, 78.6%) of fourteen patients with normal ejaculatory function showed normal ejaculatory function after surgery (preoperative MSHQ-EjD-SF vs. postoperative MSHQ-EjD-SF; 12.8 ± 2.0 vs. 12.3 ± 2.1). In the six cases of minimal urethral tearing, ejaculatory function was maintained in two of the three patients (66.7%) who had normal ejaculatory function preoperatively. In contrast, in the case of no urethral tearing, the maintenance rate of ejaculatory function at 6 months after surgery was 81.8% (9 of the 11 patients who had normal ejaculatory function preoperatively).

## 4. Discussion

The potential benefits of US-RASP make it a worthwhile option for improving the quality of life of sexually active men. Although the exact mechanisms of ejaculation dysfunction after BPH surgery are still unclear, the urethra-sparing prostatectomy technique can preserve ejaculatory function and anatomical structures, such as the prostatic urethra, bladder neck, and ejaculatory duct [13,14,16]. However, it is difficult to dissect the thin prostatic urethra when adhered to an adenoma and completely remove the adenoma. A surgical method using a robotic platform can be considered to overcome the technical difficulties of Madigan prostatectomy. John et al. successfully performed RASP in the extraperitoneal space for less ileus, a quicker return to a full diet, and less postoperative pain [5]. We implemented EUS-RASP based on the above reasons and more than 10 years of experience in performing robot-assisted radical prostatectomy. 

The US-RASP via an extraperitoneal approach was first described in a study by Wang and co-workers [11]. They performed US-RASP in patients with BPH greater than 80 mL and less than 150 mL, with a high success rate (26/27, 96.3%). They demonstrated good perioperative and functional outcomes, low urethral repair rates (7/26, 26.9%), and high maintenance rates (13/14, 92.9%) of ejaculatory function. Despite not being a comparative study, the first demonstration of the feasibility of robot-assisted Madigan prostatectomy was an encouraging finding. 

Another US-RASP study via a transperitoneal approach has suggested that the use of near-infrared fluorescence imaging in US-RASP can improve the identification and preservation of the prostatic urethra [12]. Although this study had a very small sample size (n = 12) and no comparison cohort, the results showed a low urethral injury rate of 16.6% (2/12) and an ejaculation preservation rate of 66% (8/12). Porpiglia et al. performed US-RASP in the retropubic space and compared it to the standard Millin technique RASP [14]. They also enrolled patients with a median lobe (34/92, 36%) and reported their median lobe removal technique. However, our study excluded patients with a median lobe. The urethra was completely spared in 60.86% of the cases (56/92), and minimal urethral injuries occurred in only 22.82% (21/92). A total of 83.7% (77/92) of the patients had the urethra preserved. They reported that the preservation rate of ejaculatory function increased over time and that the group with a fully preserved urethra showed a higher preservation rate of ejaculation function (89% at 12 months postoperatively). In our study, among 14 patients with preoperative antegrade ejaculation, 78.6% (11/14) maintained antegrade ejaculation 6 months postoperatively, and 81.8% (9/11) maintained antegrade ejaculation after surgery when the urethra was completely spared. In the US-RASP study described above, common results include significant improvement in postoperative micturition function, potential avoidance of CBI, and high preservation rates of ejaculation function. 

We compared and analyzed US-RASP (Madigan technique) and a nonurethra-sparing technique (Freyer approach). Both groups showed significant improvements in voiding function before and after surgery without statistical difference. The longer time until Foley catheter removal and length of hospital stay in the RASP group using the Freyer technique was likely due to the need for postoperative CBI. In the EUS-RASP group, the Foley catheter was quickly removed because CBI was not performed, which is considered to have shortened the hospitalization period. In the EUS-RASP group, manual irrigation was performed to check for intravesical clots in five patients with slightly pinkish urine, but CBI was not necessary. 

Compared to RASP, EUS-RASP preserves the urethra and bladder neck without damaging the bladder, resulting in almost no postoperative hematuria. This eliminates the need for CBI and shortens Foley catheterization, leading to less perioperative morbidity and good functional results. The urethra sparing itself using the robotic platform is sufficiently reproducible by the extraperitoneal approach, which also contributes to a rapid recovery. The most important advantage of EUS-RASP is that it allows for a high preservation rate of postoperative ejaculation.

Our study had the advantage of comparing RASP using the Freyer procedure and EUS-RASP but had several limitations. The limitations included the retrospective study design, a small sample size, and a short follow-up period. Since patients with a median lobe were excluded from the EUS-RASP group, they should be included in future studies. The failure to compare the recovery rates of voiding function and sexual function through longer follow-ups should be addressed in future studies. Nevertheless, our study has significance as a comparative study of EUS-RASP that has been under-reported to date. This study also has significance in explaining the advantages of the EUS technique. Thus, an important option in preoperative counseling for young and sexually active men with large BPH who require surgical treatment was presented.

## 5. Conclusions

EUS-RASP is a reproducible, effective, and safe surgical method compared to the existing RASP method. This technique obviated the need for postoperative CBI and shortened the time until Foley catheter removal and the hospitalization days. The introduction of this technique is expected to play an important role in improving the quality of life of sexually active male patients with large BPH by preserving ejaculation function.

## Figures and Tables

**Table 1:** Patient characteristics, preoperative BPH-related complications, and perioperative outcomes including postoperative complications according to Clavien-Dindo classification system.

Characteristics/Outcomes/Complications	EUS-RASP (n = 32)	RASP (n = 30)	*p*-Value
Characteristics			
Age	69.8 ± 6.9	71.8 ± 7.2	0.265
BMI	23.8 ± 2.3	23.6 ± 2.2	0.756
ASA score	1.9 ± 0.4	2.0 ± 0.3	0.435
Preoperative PSA (ng/mL)	8.4 ± 10.3	7.2 ± 5.8	0.565
Prostate volume (grams)	100.1 ± 20.4	99.3 ± 21.6	0.872
BPH-related complications, n (%)	14 (43.8)	17 (56.7)	0.446
Preoperative urinary retention, n (%)	10 (31.3)	14 (46.7)	0.213
Preoperative hematuria, n (%)	2 (6.3)	2 (6.7)	0.947
Preoperative UTI, n (%)	1 (3.1)	1 (3.3)	0.963
Preoperative bladder stone, n (%)	2 (6.3)	3 (10)	0.667
Preoperative hydronephrosis, n (%)	0 (0)	1 (3.3)	0.484
Preoperative bladder diverticulum, n (%)	0 (0)	1 (3.3)	0.484
Perioperative outcomes			
Operative time (min)	123.4 ± 15.2	133.7 ± 21.4	0.034
Estimated blood loss (mL)	151.3 ± 76.4	170.7 ± 104.7	0.405
Blood transfusion, n (%)	0 (0)	2 (6.7)	0.230
Change in serum hemoglobin level (g/dL)	1.5 ± 0.8	1.7 ± 0.9	0.297
Continuous bladder irrigation, n (%)	0 (0)	30 (100)	<0.001
Continuous bladder irrigation duration (days)	0	2.7 ± 0.8	<0.001
Time to Foley catheter removal (days)	2.4 ± 1.7	8.1 ± 2.4	<0.001
Time to drain removal (days)	2.0 ± 0.4	2.5 ± 0.6	<0.001
Length of hospital stay (days)	2.9 ± 1.5	4.6 ± 1.5	<0.001
Resected adenoma weight (mL)	59.4 ± 21.0	57.7 ± 21.7	0.765
Postoperative PSA (ng/mL)	1.1 ± 1.1	1.0 ± 1.0	0.882
Postoperative incontinence, n (%)	0 (0)	1 (3.3)	0.484
Urethral stricture, n (%)	0 (0)	0 (0)	NA
Follow-up duration (months)	9.9 ± 3.1	9.3 ± 3.1	0.496
Complete urethral sparing, n (%)	26/32 (81.3)	NA	
Postoperative complications, n (%)	4 (12.5)	5 (16.7)	0.728
Grade 1	1 wound infection (3.1)	0 (0)	1.000
Grade 2	3 (1 UTI, 1 mild hematuria after catheter removal, 1 delirium) (9.4)	3 (2 blood transfusions, 1 mild hematuria after catheter removal) (10)	1.000
Grade 3a	0 (0)	2 (RIH) (6.7)	0.230

Values are presented as mean ± standard deviation; BPH: benign prostatic hyperplasia; BMI: body mass index; ASA: American Society of Anesthesiologists; PSA: prostate-specific antigen; UTI: urinary tract infection; AKI: acute kidney injury; NA: non-applicable; RIH: right inguinal hernia; EUS-RASP: extraperitoneal urethra-sparing robot-assisted simple prostatectomy; RASP: robot-assisted simple prostatectomy.

**Table 2:** Functional data.

Variables	EUS-RASP (n = 32)	RASP (n = 30)	*p*-Value
IPSS			
Baseline	22.3 ± 6.8	23.8 ± 6.1	0.369
6 month	6.4 ± 2.6	7.2 ± 2.8	0.252
IPSS decrease	16.0 ± 6.7	16.7 ± 5.6	0.659
IPSS-QOL			
Baseline	4.3 ± 1.1	4.6 ± 1.0	0.226
6 month	1.5 ± 1.1	1.7 ± 1.0	0.443
IPSS-QOL decrease	2.8 ± 1.4	2.9 ± 1.2	0.720
Maximum urinary flow rate (mL/s)			
Baseline	6.6 ± 3.9	5.9 ± 3.1	0.439
6 month	19.4 ± 6.4	17.1 ± 6.0	0.159
Qmax increase	12.8 ± 5.8	11.3 ± 5.8	0.302
Post-void residual volume (mL)			
Baseline	141.8 ± 155.8	153.5 ± 135.4	0.753
6 month	11.1 ± 9.2	16.9 ± 16.9	0.104
PVR decrease	130.6 ± 153.3	136.6 ± 130.3	0.870
Sexual function			
IIEF-5 of total 32 patients			
Baseline	15.3 ± 7.8		
6 month	15.0 ± 7.8		
IIEF-5 more than 17 point preoperatively	18/32 (56.3)		
Baseline	20.4 ± 2.1	NA	NA
6 month	20.1 ± 2.1	NA	NA
MSHQ-EJD-SF of total patients			
Baseline	9.1 ± 4.5	NA	NA
6 month	7.9 ± 4.5	NA	NA
MSHQ-EJD-SF; preoperative normal EF	14/32 (43.8)		
Baseline	12.8 ± 2.0	NA	NA
6 month	12.3 ± 2.1	NA	NA
Ejaculatory function			
Preoperatively normal EF, n (%)	14/32 (43.8)	NA	NA
Postoperative antegrade ejaculation, n (%)	11/14 (78.6)	NA	NA
Postoperative antegrade ejaculation of patients with postoperative intact urethra, n (%)	9/11 (81.8)	NA	NA

Values are presented as mean ± standard deviation. IPSS: International Prostate Symptom Score; QoL: quality of life; Qmax: maximal urinary flow rate; PVR: Post-void residual volume; IIEF: International Index of Erectile Function; MSHQ-EjD-SF: Male Sexual Health Questionnaire Ejaculatory Dysfunction Short Form (range 0–15); EF: ejaculatory function; NA: non-applicable; EUS-RASP: extraperitoneal urethra-sparing robot-assisted simple prostatectomy; RASP: robot-assisted simple prostatectomy.

## Data Availability

The data presented in this study are available upon request.
